# Recent Advances in the Separation of Rare Earth Elements Using Mesoporous Hybrid Materials

**DOI:** 10.1002/tcr.201800012

**Published:** 2018-05-27

**Authors:** Yimu Hu, Justyna Florek, Dominic Larivière, Frédéric‐Georges Fontaine, Freddy Kleitz

**Affiliations:** ^1^ Department of Chemistry Université Laval Québec G1V 0A6, QC Canada; ^2^ Centre en Catalyse et Chimie Verte (C3V) Université Laval, Québec G1V 0A6, QC Canada; ^3^ Department of Inorganic Chemistry – Functional Materials, Faculty of Chemistry University of Vienna 1090 Vienna Austria; ^4^ Canada Research Chair in Green Catalysis and Metal-Free Processes

**Keywords:** Rare earth elements, critical metals, solid-phase extraction, adsorption, mesoporous materials, hybrid sorbents, chelating ligands

## Abstract

Over the past decades, the need for rare earth elements (REEs) has increased substantially, mostly because these elements are used as valuable additives in advanced technologies. However, the difference in ionic radius between neighboring REEs is small, which renders an efficient sized‐based separation extremely challenging. Among different types of extraction methods, solid‐phase extraction (SPE) is a promising candidate, featuring high enrichment factor, rapid adsorption kinetics, reduced solvent consumption and minimized waste generation. The great challenge remains yet to develop highly efficient and selective adsorbents for this process. In this regard, ordered mesoporous materials (OMMs) possess high specific surface area, tunable pore size, large pore volume, as well as stable and interconnected frameworks with active pore surfaces for functionalization. Such features meet the requirements for enhanced adsorbents, not only providing huge reactional interface and large surface capable of accommodating guest species, but also enabling the possibility of ion‐specific binding for enrichment and separation purposes. This short personal account summarizes some of the recent advances in the use of porous hybrid materials as selective sorbents for REE separation and purification, with particular attention devoted to ordered mesoporous silica and carbon‐based sorbents.

## 1 Introduction – The Need of New Sorbents for Rare Earth Separation

The rare earth elements (REEs), as defined by IUPAC, are a group of 17 elements including 15 lanthanides (Ln), scandium (Sc) and yttrium (Y). The importance of REEs is booming owing to their unique electrical, magnetic, and optical properties and their wide applications in electronics, optics, metallurgy, and other advanced fields.^1^,^2^ For example, the demands for REEs has drastically increased during the past few decades, mostly because of their use in low‐carbon and green energy technologies, such as electric vehicles (Dy and La), wind power (Nd), and energy‐efficient lighting (La, Gd, Tb, and Eu). The main applications of REEs are compiled in Table 1.^3^

**Table 1:** The rare earth elements (REEs) and some of their main applications.^3^

Element	Symbol	Applications
Scandium	Sc	Metal alloys for the aerospace industry
Yttrium	Y	Capacitors, metal alloys, lasers, sensors, superconductors
Lanthanum	La	Ceramics, batteries, car catalysts, phosphors, pigments, X‐ray
Cerium	Ce	Catalysts, polishing, metal alloys, UV filters
Praseodymium	Pr	Pigments, lightning, lenses, glasses
Neodymium	Nd	Permanent magnets, lasers, catalysts, infrared filters
Promethium	Pm	Beta radiation source, fluid‐fracking catalysts, phosphors
Samarium	Sm	High‐temperature magnets; nuclear reactor control rods
Europium	Eu	Liquid crystal displays, fluorescent lighting, glass additives, phosphors
Gadolinium	Gd	Magnetic resonance imaging contrast agent, glass additives
Terbium	Tb	Phosphors, electronics
Dysprosium	Dy	High‐power magnets, lasers, guidance systems
Holmium	Ho	High‐power magnets, nuclear industry
Erbium	Er	Lasers, glass colorant, optical fibers, ceramics
Thulium	Tm	High‐power magnets
Ytterbium	Yb	Fiber‐optic technology, solar panels, alloys, lasers, radiation source for portable X‐ray units
Lutetium	Lu	X‐ray phosphors, single crystal scintillators

Contrary to their name, REEs are moderately abundant in the earth's crust; however, they are rarely found in *easy‐to‐mine* minerals, and are often unfavorably distributed in common ores/minerals.^1^ The recent reduction in the exportation quotas from China ‐ which produces more than 90 % of the world's supply ‐ has also increased the price of REEs, making mining prospects for these elements more compelling. Because of their importance and restricted supplies, REEs belong to the group of critical metals. However, most of the flow sheets for the separation of REEs are not environmentally friendly, and the mining of REEs in order to meet the growing global demand has caused perceptible environmental damage. As a result, the need for alternative sources of supply and eco‐friendly purification processes has risen. Among the various envisioned possibilities, the recovery of REEs from industrial and mining waste and end‐of‐life products (e. g., electronics, optics, and magnets) represents an economically and environmentally attractive and viable approach.

Most REEs have a stable d^0^ electronic configuration by forming trivalent cations (+III, except for a few REEs in specific redox and pH conditions). Even if the various ions have similar physicochemical properties, a gradual decrease in ionic radius is observed as a function of increasing atomic number (ranging from 86.1 pm for Lu^3+^ to 103.2 pm for La^3+^, 90.0 pm for Y^3+^, and 74.5 pm for Sc^3+^).^4^ The physicochemical similarity between the elements is also one of the biggest obstacles hampering the selective extraction of REEs. Hydrometallurgical approaches, including chemical precipitation, liquid‐liquid extraction (LLE), resin‐based supported‐liquid extraction (SLE), solid‐phase extraction (SPE), ion‐exchange, and super critical fluid extraction, are common chemical extraction methods of separating individual rare earth oxides (REOs) from the mineral concentrate.^3^ The LLE, which largely dominates the purification process nowadays, has limited selectivity among adjacent elements and therefore consumes a large amount of organic solvent during repetitive extractions cycles, thus generating undesired and harmful waste.^5^, ^6^, ^7^ On the other hand, the leaching of the extracting functional groups into the aqueous phase is inevitable in SLE, since the ligand is only physically impregnated on the resin, which results in low reusability and high cost.^8^,^9^ In comparison, the emerging SPE systems require less solvent, provide high enrichment factors, and reduce the risk of cross contamination, and thus are a promising sample treatment technique. In SPE, the chemical anchoring of the functional groups on the support through covalent bonds ensures greater regenerative capacities, ultimately reducing the operating cost. Besides the complexation features resulting from the grafting of the ligands, the capacity and efficiency of the SPE systems is also determined by the characteristics of the solid supports (adsorbents).^10^,^11^ In particular, ordered mesoporous materials (OMMs) (namely, ordered mesoporous MCM/SBA‐type silicas and carbons) possess high specific surface area, high pore volume, and anchoring sites that are available to covalently bind various organic ligands to the surface, yielding materials with high functionality, stability, and enhanced regeneration abilities.^12^, ^13^, ^14^ This contribution highlights some of the recent progress achieved in the development of SPE materials for efficient and selective separation of REEs, especially using mesoporous sorbents as solid supports. By choice, this account emphasizes especially on the authors’ own contributions in the area, with the addition of other pertinent references.

## 2 Short Overview of the Industrial REE Extraction Process: Principle, Methodology and Challenges

**Figure 1:**
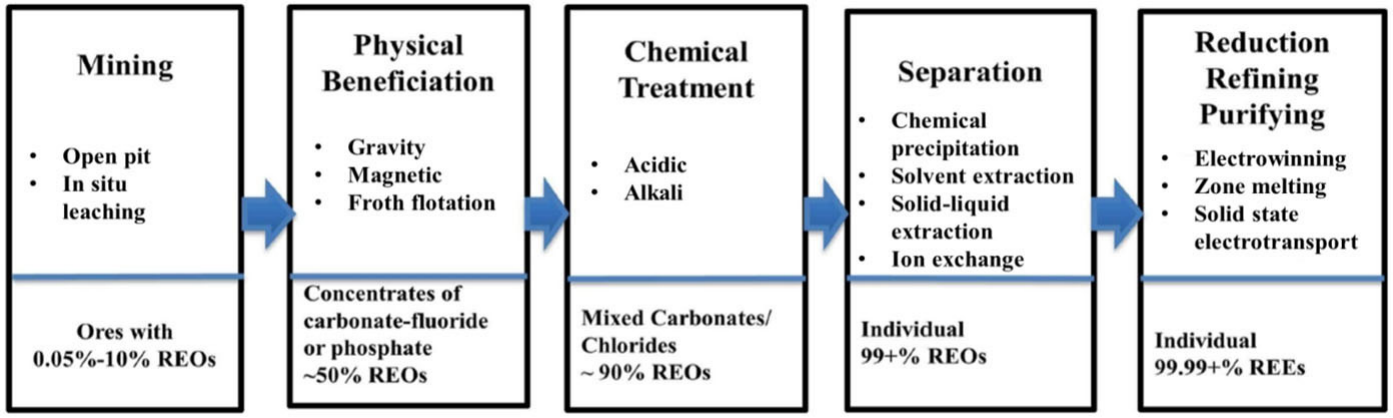
General processing routes for REE ores. Reproduced and adapted with permission from ref [15]. Copyright 2014, Frontiers Media.

The REEs are found in a variety of minerals. Due to the complex matrices associated with those minerals, several processing steps are required in order to physically and chemically break down the minerals containing the REEs. In general, the REE processing route includes the following major steps: mining, beneficiation, chemical treatment, separation, reduction, refining, and purification (Figure 1).^15^ The REE mineral concentrate, which contains approximately 50 % rare earth oxides (REOs), is obtained from mining the ores and physically separated from gauge minerals. The physical beneficiation processes, each adapted to different types of minerals and ores, include grinding, sifting, gravitational separation, magnetic separation, and froth flotation. These treatments generate REEs in the form of fluorocarbonates and phosphates. The following chemical treatment step converts REE minerals into carbonates or chlorides through hydrometallurgy processes, in which the REOs are exposed to strong acid (HF, HCl, H_2_SO_4_) or base (NaOH, Na_2_CO_3_). However, the leaching steps are seldom selective, and large amounts of competing elements can be extracted.^16^ An additional difficulty arises from the unfavorable distributions (concentrations) of distinct rare earth metals in common ores/minerals. Therefore, supplementary separation and purification steps are required in order to obtain the REEs with satisfying purity for further advanced applications (steps four and five in Figure 1).

**Figure 2:**
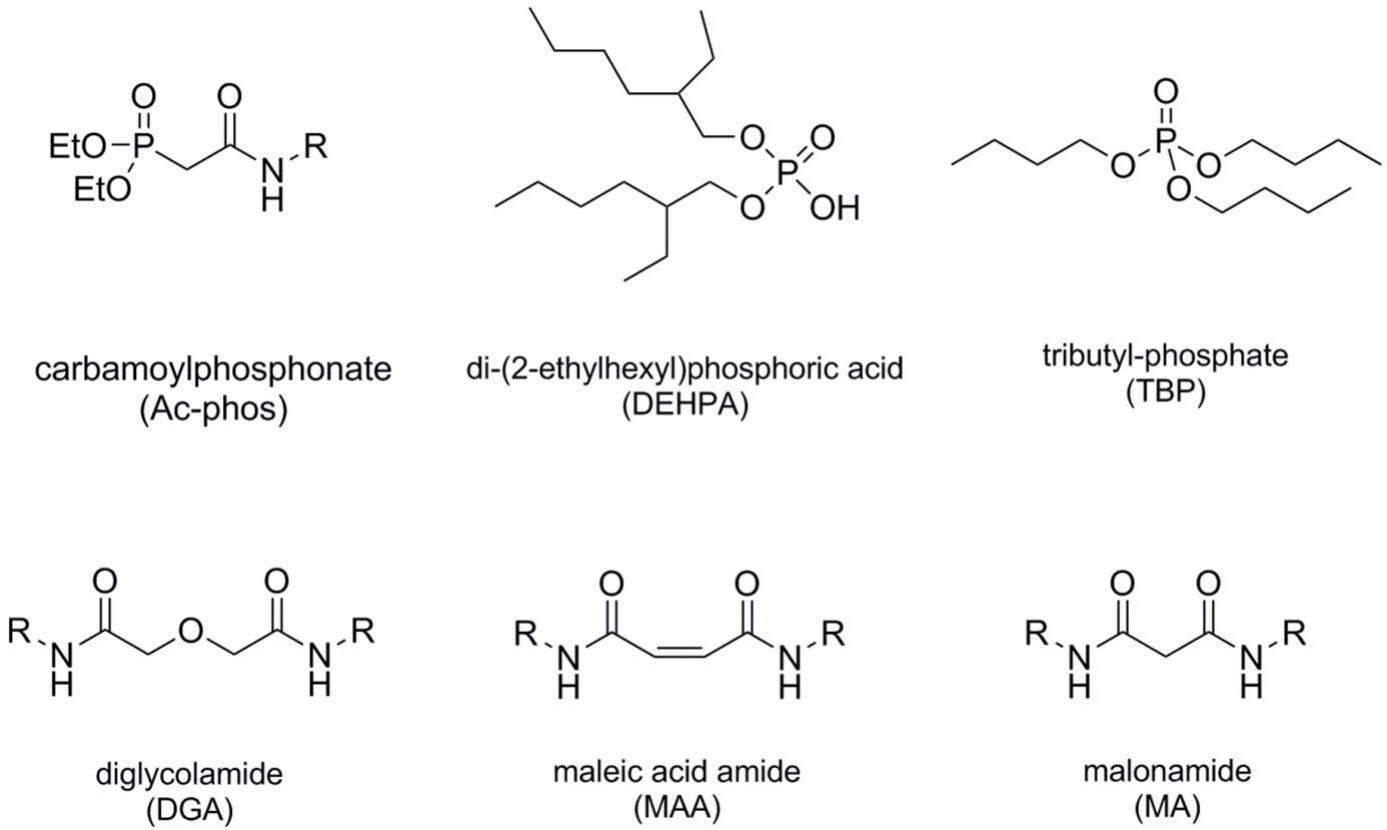
Some of the ligands that are commonly used in industrial liquid‐liquid extraction (LLE) of REEs.

As mentioned above, the conventional processes for the separation of REEs mainly include chemical precipitation,^17^, ^18^, ^19^ solvent extraction (or liquid‐liquid extraction, LLE),^20^ ion exchange,^6^ and solid‐liquid extraction, i. e., supported‐liquid extraction (SLE) and solid‐phase extraction (SPE) methods, which rely on the association between organic ligands and REEs.^21^ Ion exchange can be used to obtain REEs with purities higher than 99.9999 %; however, its large‐scale application is limited by the high cost.^6^ Nowadays, to retrieve individual REEs, multi‐stage LLE is applied on the industrial level. The recent developments of the LLE process focus on the optimization of selective extractants and organic solvents in order to improve the separation efficiency and enrichment factors, as summarized by Verboom *et al*. in their recent review.^20^ It can be generalized that the high Lewis acidity of Ln^3+^ favors the coordination with hard nucleophilic organic ligands to form stable complexes. Currently, most of the extractants used in industrial LLE of REEs contain oxygen, nitrogen, sulfur and/or phosphorous atoms, with the most effective ones being oxygen‐donor chelating ligands. Some examples are shown in the Figure 2. However, LLE is frequently plagued by practical problems such as slow extraction kinetics, the low solubility of some extractants in aliphatic diluents,^22^ and the formation of emulsion.^7^ Furthermore, a large number of extractants used in LLE suffer from poor selectivity among adjacent elements, thus consuming large volumes of high‐purity solvents upon repetitive extraction cycles, and generating a large amount of undesired and radioactive wastes. A life‐cycle assessment of the production of REEs shows that mining, leaching and solvent extraction have the largest contribution to the overall environmental footprint.^23^ This adverse impact caused by LLE contradicts the green chemistry and clean energy principles and could overshadow the potential of technologies relying on REEs. Ionic liquid (ILs) are currently being investigated as alternative extraction media to conventional organic solvent in LLE; however, this separation approach is restricted by the high viscosity and the low solubility of ILs, and by the difficulty of recovering the metal species due to the strong interactions between ILs and the targeted metals.^24^,^25^

In comparison, solid‐liquid extraction (SLE and SPE) is a greener technique for element extraction/separation than LLE, in part because it exploits the large surface area properties of highly porous materials. Both SLE and SPE use the affinity of a flowing liquid containing the dissolved or suspended analytes (known as the mobile phase) with a solid (known as the stationary phase), which leads to the separation of the mixture into desired and undesired fractions of the components. Compared to conventional LLE, these techniques feature a higher enrichment factor and a faster phase separation, and drastically reduce the consumption of solvent and the production of pollutants. In SLE, a REE‐selective ligand is dissolved in a hydrophobic organic phase which is physically impregnated or coated on the solid support (e. g., a polymer resin), and the aqueous phase is then passed through the column (or cartridge) where the extraction takes place. Unfortunately, the wet impregnation strategies often result in the pronounced stripping/leaching of the stationary liquid phase, causing cross‐contamination and limited lifetime, thus hampering their applicability. To overcome these issues, SPE has been proposed as a cost‐effective alternative, in which an extracting agent is chemically anchored on a solid support. In this context, materials containing nanosize pores (i. e., nanopores), in particular ordered mesoporous silica (OMS) and carbon (OMC) materials, have received great attention as promising candidates for solid supports, because of their superior extraction capacity, stability, and possibility of functionalization,^26^ as it will be elaborated in the following sections. In SPE, the extraction performance of the sorbents is usually quantified by the distribution coefficient K_d_ values (mL g^−1^) that are calculated by the following equation:

$$K_{d}=\frac{V}{m}\times \frac{C_{0}-C_{f}}{C_{f}}$$

where V is the volume of the solution, m is the amount of the sorbents, and C_0_ and C_f_ are the initial and final concentration of the metal ions, respectively.

## 3 Requirements for SPE Adsorbents, and the Advantages of Using Ordered Mesoporous Materials

**Figure 3:**
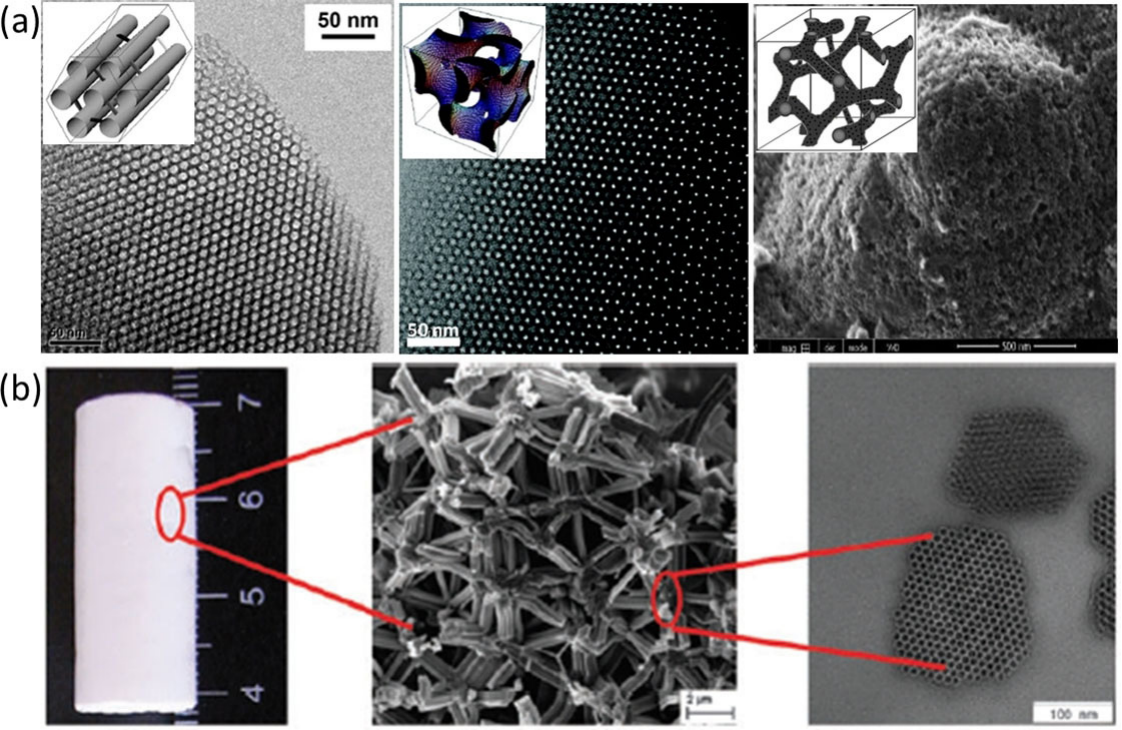
(a) TEM/SEM images of ordered mesoporous materials and the schematic representation of their mesostructure (insert) (from left to right: SBA‐15 or MCM‐41, KIT‐6, and CMK‐8), and (b) Photograph and SEM/TEM images of a silica monolith exhibiting two or more separated pore size regimes. Reproduced with permission from ref [28], [30], [39], and [40]. Copyright 2010, ACS, 2017, ACS, and 2013, RSC.

In principle, the SPE procedure is based on the adsorption of the desired species onto the surface of a given adsorbent; therefore, the overall performance of the adsorbents can be optimized by tuning the characteristics of both the organic ligand and the solid support. In SPE, a good solid support should meet the following requirements: (1) it should have a large surface area in order to achieve high extraction capacity, (2) it should be easily modifiable with functional groups, (3) it should provide the possibility of shape control to adjust the materials to various applications, and (4) be robust and reusable. Earlier studies have focused on the use of traditional porous materials, such as bare silica gels^27^ and activated carbon,^28^ as solid supports. The development of advanced adsorbents bearing multi‐functions and improved performance has become a continuing object of research, and the emerging nanoporous materials (pore size <100 nm) have been widely tested as potential practical adsorbents because of their high intrinsic specific surface area. Based on their pore sizes, nanoporous materials can also be classified into four types: microporous (<2 nm), mesoporous (2–50 nm), macroporous (>50 nm), and hierarchically porous materials which combine two or three of the above pore size ranges.^13^ In the case of silica‐based mesoporous materials, some of the most interesting materials, both from the industrial and fundamental research points of view, are ordered mesoporous silica (OMS) materials with a well‐defined pore network such as MCM‐41 and SBA‐15 (OMS with hexagonal array of cylindrical pores, with pore size of 2–15 nm),^29^, ^30^, ^31^ KIT‐6 (OMS with highly interconnected 3‐D cubic structure),^32^,^33^ and bimodal mesoporous‐macroporous silica with hierarchical architectural properties (Figure 3).^34^,^35^ Cooperative assembly between “soft” organic templates (e. g., triblock copolymers, quaternary cationic surfactants, C_n_H_2n+1_N(CH_3_)_3_Br (n=8‐22)) and inorganic precursors (e. g., tetraethylorthosilicate (TEOS), tetramethylorthosilicate (TMOS)) in either acidic or basic media is generally involved, forming inorganic/organic mesostructured composites.^13^ After template removal, the resulting nanoporosity of these materials ensures their particularly high specific surface area (typically >800–1000 m^2^ g^−1^), which undoubtedly allows for a high degree of contact and functionalization. The pore size, pore shape, pore connectivity, and wall thickness of silica materials can be modulated precisely as a function of the synthesis parameters, such as choice of the template (e. g., surfactant molecule with different chain length), solution pH, hydrothermal treatment conditions (temperature and time), and addition of secondary structure‐directing agents such as co‐surfactant, swelling agents, and electrolytes.^13^,^31^,^33^,^36^, ^37^, ^38^ Another interesting aspect of these materials is the possibility to synthesize hierarchically structured silica‐based porous monoliths *via* sol‐gel processes allowing versatility both in terms of size and morphology (shape), which should be particularly beneficial for industrial applications.^39^,^40^ Furthermore, silica materials are considered fairly stable in the pH range commonly used for REE extraction, i. e., pH 3 to 8, and are environmentally benign.

The incorporation of functional groups onto a silica surface or into the pore walls can mainly be achieved in three ways: 1) by subsequent reaction of organosilanes of the type (R'O)_3_SiR with the free silanol groups on the pore surfaces (grafting), 2) by co‐condensation of tetraalkoxysilanes (TEOS or TMOS) with terminal trialkoxyorganosilanes of the type (R'O)_3_SiR in the presence of structure‐directing agents (one‐pot synthesis), and 3) by hydrolysis and condensation reactions of bridged organosilanes of the type (R'O)_3_SiRSi(OR’)_3_ leading to periodic mesoporous organosilicas (PMOs).^12^ The wide abundance of the silanol groups (i. e., in average 1–2 Si‐OH nm^−2^)^43^ allows for the efficient and easy functionalization through condensation reactions, producing siloxane species (i. e., grafting procedure).^12^,^14^ Thus, it is so far the most commonly applied method for the synthesis of functionalized materials for REE extraction. However, in the case of materials with small pore size or narrow pore entrance, or when the grafted species are very bulky, the organosilanes may react preferentially at the pore openings during the initial stages, thus leading to a nonhomogeneous distribution of the organic groups within the pores. In comparison, co‐condensation synthesis can effectively overcome the “pore blocking” problem and the heterogeneous distribution of organic units. In REE extraction, the co‐condensation pathway has frequently been applied for the synthesis of metal ion‐imprinted mesoporous silica (IMS) with highly ordered structures (see below). Finally, since the organic bridges are integral components of the silica network, the PMOs are in general characterized by a periodically organized pore system and a narrow pore size distribution. However, synthesis of high quality PMOs with high content of organic groups is sometimes difficult to achieve.

Ordered mesoporous carbon (OMC) materials, on the other hand, may be ideal candidates as solid supports under acidic conditions as they usually possess higher chemical resistance. However, the synthesis and the functionalization of carbon materials are more challenging than for silica materials. In general, highly ordered mesoporous carbon can be synthesized by either hard templating (i. e., nanocasting) or soft templating methods (through the combination of phenolic‐type resins and triblock copolymer templates).^44^,^45^ The nanocasting route, in which porous silicas are used as solid templates before being removed by strong base (NaOH) or acid (HF), yields CMK‐type materials with a very high surface area (higher than 2000 m^2^ g^−1^ for some of the nanocasts) and a well‐structured mesopore network (e. g., CMK‐8 as a model of high‐surface area ordered mesoporous carbon structure, Figure 3).^41^ The mesostructure of the final OMC can easily be tuned by modifying the characteristics of the hard silica template. In the one‐pot, soft‐templating method, the carbon mesophases are prepared by a similar way to the silica analogues, and the porosity and morphology can be controlled by varying the synthesis conditions, such as pH, temperature, and gel composition.^45^

Parallel to the intensive studies of ordered mesoporous materials, systems exhibiting a disordered structure were also reported.^46^, ^47^, ^48^ However, the disordered structure often leads to the co‐existence of small and large pores and wide pore size distribution. Disordered porous materials with narrow pore size distribution were reported (e. g., controlled‐pore glass, CPG);^49^,^50^ yet their applications in ion adsorption remain scarce. In comparison, ordered mesoporous materials possess well‐calibrated parameters in terms of pore size and pore size distribution, which ensures high specific surface area and uniform pore environment, while it also facilitates the characterization of the sorbents. Therefore, in this work, we focus on the recent development of REE extraction systems based on ordered mesoporous materials.

## 4 Recent Progress in SPE of REEs Using Functionalized Mesoporous Materials

### 4.1 Ordered Mesoporous Silica (OMS)

As mentioned above, the choice of using mesoporous silica materials (e. g., MCM‐41, SBA‐15, and KIT‐6) is motivated by their high porosity allowing for a substantially enhanced adsorption capacity and a high contact efficiency, and the abundance of silanol groups on the pore surface enabling easy functionalization procedures (with ligands) through chemical grafting. However, the influence of the silanol groups present in the pristine silica materials should not be neglected. For example, high uranium (UO_2_^2+^) physisorption on bare silica (e. g., SBA‐15 and MSU‐H) has been previously observed.^51^,^52^ Similarly, Giret *et al*. demonstrated the easy use of non‐functionalized silica‐based mesoporous materials for the separation and enrichment of Sc^3+^.^53^ Several mesoporous silica‐based materials under investigation (namely, KIT‐6, SBA‐15, and silica gel) were shown to exhibit an exceedingly high level of preconcentration for Sc^3+^, with KIT‐6 showing the highest distribution coefficient value (K_d_=950 mL g^−1^) and extraction capacity (1 mg g^−1^), possibly owing to its interconnected 3‐D pore structure. In acidic conditions, extraction yields exceeding 90 % were achieved. The selectivity with respect to other REEs was assessed, with Sc^3+^ being the sole REE retained in a chromatographic mode. Here, the selective extraction of Sc^3+^ over the other elements (lanthanides, Al^3+^, Fe^3+^) could be explained by the presence of interactions with the abundant surface silanol groups in the KIT‐6 silica. Furthermore, a simple yet effective separation scheme based on the KIT‐6 sorbent was designed and tested for the Sc^3+^ extraction from ore leachates, and an enrichment of more than 11000 % was achieved. Therefore, the presence of free silanol groups on the sorbents after ligand grafting should also be taken into consideration when studying the extraction behavior of functionalized mesoporous materials. Nevertheless, there are only few reports of using bare silica for the separation of critical metals, and the introduction (*via* grafting or co‐condensation) of selective ligands is mostly required, as discussed below.

**Figure 4:**
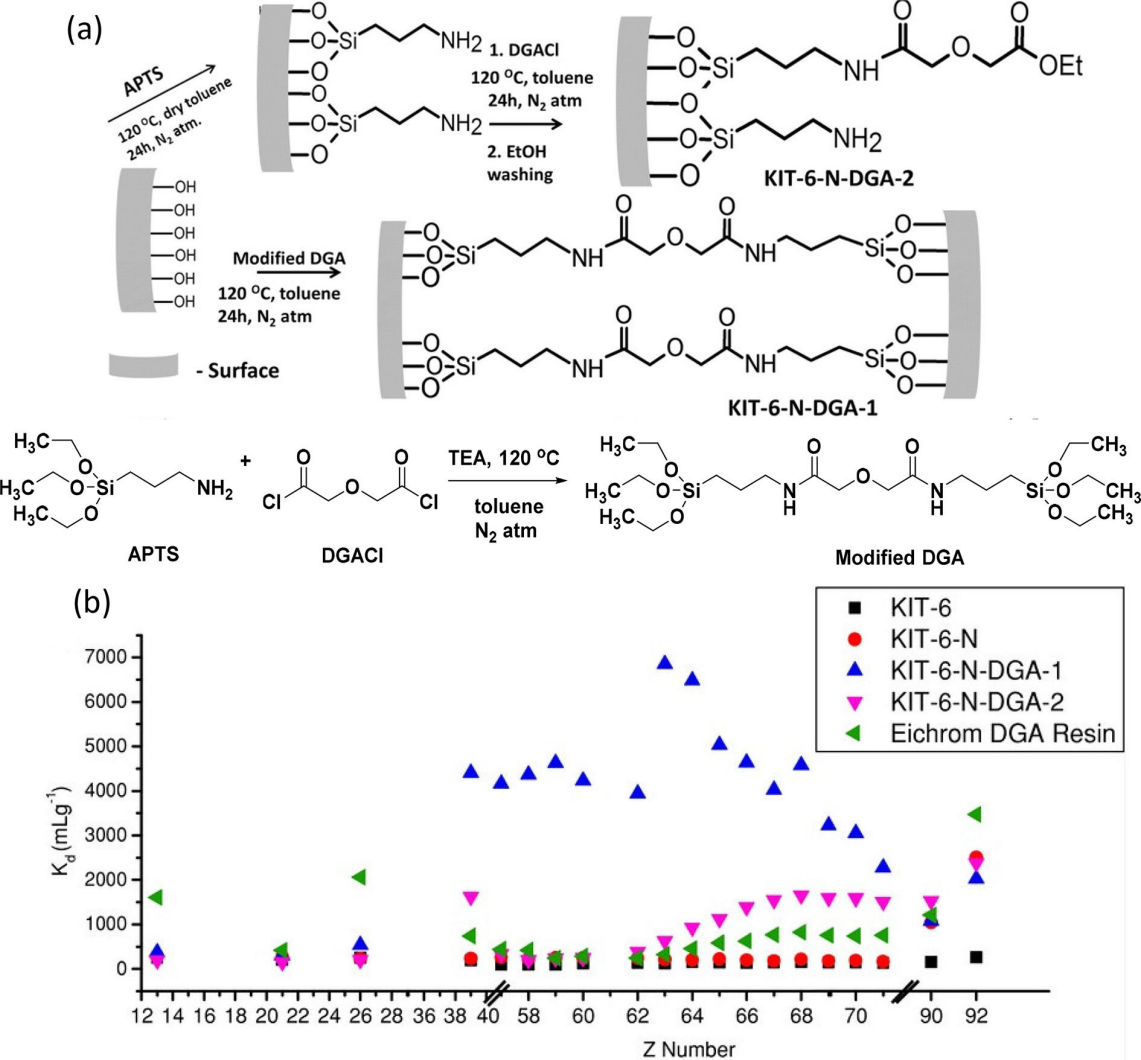
(a) One‐step and two‐step modifications of the surface of KIT‐6 silica to generate the mesoporous REE sorbents. (b) Extraction capacity for REEs in the presence of competitive ions (Al^3+^, Fe^3+^, Th^4+^ and UO_2_^2+^). Reproduced with permission from ref [56]. Copyright 2014, Wiley.

Initially, most of the sorbents developed for REE separation are functionalized with well‐known ligands used in LLE applications. Fryxell and coworkers, for example, pioneered the synthesis of functional self‐assembled monolayers on mesoporous supports (SAMMS) using phosphonic acid and phosphoric ester derivatives, which show generally good extraction behavior for REEs in LLE systems (Figure 2).^54^ The ester‐modified material showed a high retention of competing ions (i. e., Fe^3+^, Ni^2+^, Cu^2+^, Zn^2+^, K^+^, and Ca^2+^) compared to the targeted REEs, whereas the acid‐functionalized SAMMS material showed a significant sorption selectivity towards lanthanides. Following these early reports, our team studied the functionalization of large pore (>5 nm) ordered mesoporous silica materials, e. g., SBA‐15 and KIT‐6, for the selective extraction of actinides, such as UO_2_^2+^ and Th^4+^.^55^ It appeared that phosphonate‐functionalized KIT‐6 hybrid materials exhibit excellent sorption capacity, interesting selectivity in multi‐element mixtures, and good stability enabling regeneration and reusability over several cycles. Inspired by this success, we initiated a program to develop more efficient and recyclable REE sorbents using similar silica supports. Focusing more on nitrogen‐containing ligands rather than phosphorous‐based ones, we decided to immobilise the diglycolamide (DGA) ligand on large pore mesoporous silica and use it as a REE separation sorbent in an SPE system.^56^ The DGA is well known to exhibit high extraction capacity and a selectivity for heavy REEs in LLE.^57^ In order to investigate the effect of the grafting procedure on the behavior of functionalized materials, the DGA ligand was grafted on KIT‐6 silica either in a one‐step (KIT‐6‐N‐DGA‐1) or a two‐step (KIT‐6‐N‐DGA‐2) sequence (Figure 4a), while the commercial DGA‐based resin was used as a reference. In the case of the one‐step grafting procedure, a pre‐synthesized DGA‐bridged disilane (i. e., modified DGA in Figure 4a) was directly grafted on the silica mesopores through a condensation reaction with the silanol groups, while in the two‐step functionalization process, an aminopropyl chain was first attached to the silica surface, followed by reaction with diglycolyl chloride. The extraction tests showed that the KIT‐6‐N‐DGA‐1 exhibits an extraction capacity significantly enhanced as well as an increased selectivity for middle‐sized REEs compared to both KIT‐6‐N‐DGA‐2 and the commercial DGA resin (Figure 4b). It is obvious that different extraction performances are achieved by varying the grafting protocol of a same chelating ligand. In this example, we demonstrated that a two‐step grafting leads to a mixture of different functionalities with the ligand attached on the surface by either one or two amino moieties, leading to the absence of size specificity and poorer selectivity. For KIT‐6‐N‐DGA‐1, the selectivity for complexing trivalent metal ions stems from the favorable geometry of the cavity formed by the organic functionalities grafted on the solid support by both end groups. We suggested that the increased rigidity in the chelating ligand anchored on the surface by both sides reduces the *coordination flexibility*, resulting in a size‐specific cavity, in contrast to solution chemistry. Moreover, it is believed that the large pore size of KIT‐6‐type materials reduces the risk of pore blocking and is beneficial for the diffusion of the metal ions (ion radius in the range of 80–100 pm for REEs, plus hydration sphere) through the system. The adsorbed REE ions can be removed using a (NH_4_)_2_C_2_O_4_ solution (stripping), and the functionalized materials obtained through the grafting procedure are reusable for up to 5 adsorption‐stripping‐regeneration cycles, showing their marketable potential. The stability of the organic moieties after extraction was also confirmed by thermogravimetric analysis (TGA) and solid‐state NMR spectroscopy, as no significant change in the integrity of the organic units was observed.

Following these first studies, Zheng *et al*. reported a similar silica system where maleic anhydride is attached on the surface of mesoporous silica nanoparticles.^58^ Corroborating the results published by our group, the synthesized sorbents demonstrated higher selectivity for Sm^3+^, Eu^3+^, and Gd^3+^ (i. e., the SEG group), and lower uptake for competing elements was observed. Our group further focused on the investigation of the role of the silica support on the extraction efficiency, in particular under dynamic flow‐through conditions. In a subsequent study, the role of parameters such as materials’ pore size (3–10 nm), pore network structure (2‐D *vs* 3‐D) and pore shape (cylindrical *vs* cage‐like) was investigated for the DGA‐grafted system.^59^ The results showed that SBA‐16 silica is more prone to pore blocking due to its cage‐like pore structure and narrow connectivity of the pores, which lead to a reduced accessibility to the coordinating sites of the DGA ligand. For the SBA‐15 materials with a cylindrical pore shape, the size of the pore (5–10 nm) was tuned by varying the aging temperature during synthesis (60–130 °C). These results showed that a pore size between 5–8 nm (i. e., SBA‐15 aged at a temperature below 100 °C) provides a certain confinement of the targeted ions and therefore enhanced the REE adsorption, whereas smaller pore size (MCM‐41 with pore size <4 nm) can easily result in the obstruction of the entrance of the pores during the grafting, thus hampering the extraction capacity. These findings provided insights into the contributions of porosity and pore morphology of the functionalized solid supports for the material design, both in batch and dynamic flow‐through systems.

**Figure 5:**
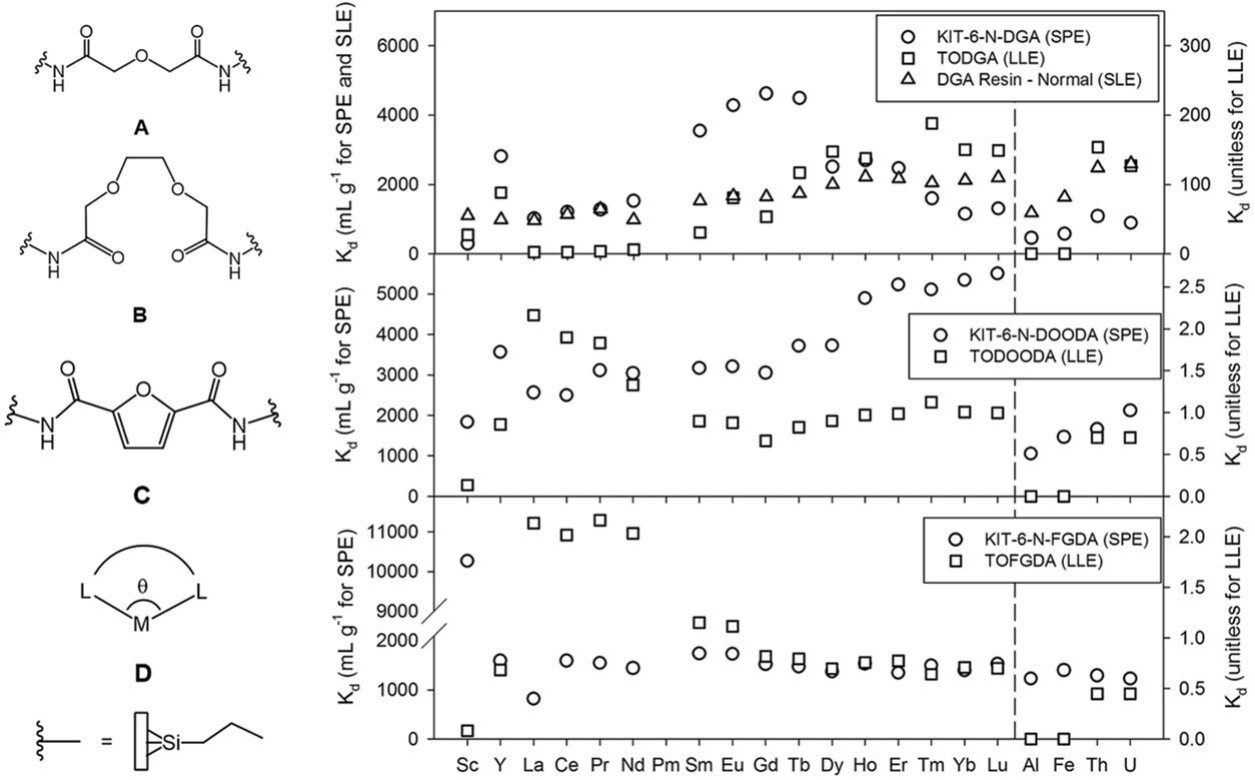
(A) DGA, (B) DOODA and (C) FDGA ligands grafted on KIT‐6 silica, and the corresponding distribution coefficient (K_d_) values for SPE (left scale) compared to LLE and SLE counterparts (right scale). Reproduced with permission from ref [60]. Copyright 2015, RSC.

**Figure 6:**
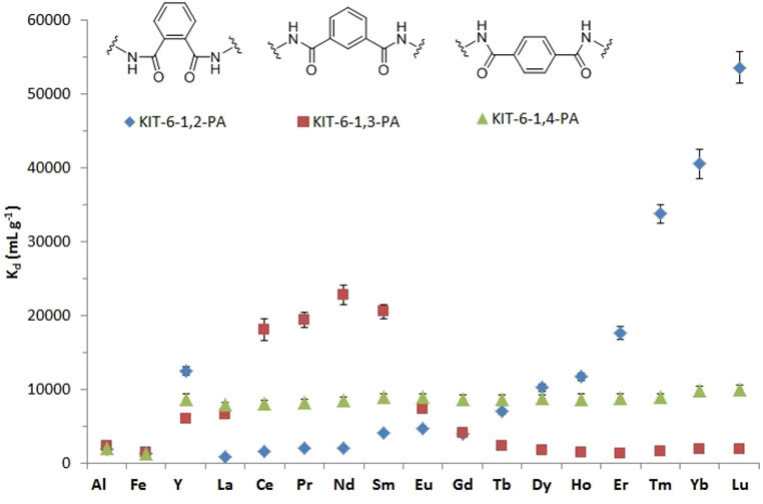
Distribution coefficient (K_d_) values for phthaloyl diamide (PA)‐functionalized hybrid materials. Reproduced with permission from ref [64]. Copyright 2017, ACS.

From a chemical point of view, the design of ligands with superior selectivity also plays a critical role in enhancing the overall extraction performance of sorbents. The Ln^3+^ cations are highly Lewis acidic and easily coordinate nucleophiles to form stable complexes. The angle formed by chelate ligands, which could be described as the *bite angle* in a certain analogy to d‐metal complexes (Figure 5D), can greatly affect the binding properties of these ligands. In theory, ligands with larger *bite angles* will have a higher affinity for larger ions whereas smaller bite angles will favor coordination to smaller ions. In another report, it was evidenced that by tuning the *bite angle*, a certain degree of selectivity towards different groups of REEs can be achieved. For example, the derivative of 3,6‐dioxaoctanediamide (DOODA) has a smaller *bite angle* than the DGA ligand, and thus the DOODA‐modified mesoporous material shows a preference for extracting smaller lanthanides (Figure 6B).^60^ On the other hand, a ligand bearing a larger *bite angle*, such as furan‐2,4‐diamide (FDGA), shows unexpected high affinity toward Sc^3+^ in SPE. Most importantly, when compared to their homogeneous counterparts used in LLE systems, a distinctly different extraction behavior (in terms of selectivity and extraction capacity) was observed for SPE. This different behavior further demonstrates the importance of ligand grafting in order to improve the extraction performance of SPE systems. The immobilization of a ligand on the mesoporous surface lowers its flexibility and provides a more stable *bite angle*, yielding a more pronounced selectivity towards selected REE cations.

It has become obvious that the design of new ligands in order to control the nature of the coordination cavity generated when anchored onto a solid surface could be of great interest to achieve further selectivity for critical metals. However, the rotation of the σ−σ bond in DGA and DOODA in the sorbents described above might have an adverse effect on the overall rigidity of the hybrid materials, thus hampering their selectivity. Under this context, the concept of “ligand preorganization” was brought to attention. With a multidentate, preorganized ligand, donor atoms are selectively arranged in order to achieve favorable interactions with a selected metal ion. To the best of our knowledge, most of the preorganized ligands to date are mixed soft N‐donor and hard O‐donor extractants for the selective enrichment of actinides,^61^, ^62^, ^63^ while successful examples of highly rigidified sorbents for REEs are still scarce. To follow this direction, we grafted on KIT‐6 silica a series of preorganized ligands based on phthaloyl diamide (PA). Different *bite angles* were formed by the *ortho*, *meta*, and *para* positioning of the amide groups, and the materials were tested as sorbents for REEs.^64^ The conjugated aromatic structure would provide rigidity, making phthaloyl diamide preorganized for forming complexes with REEs of different ionic radii. In these systems, it is expected that the silanol groups and the siloxane oxygen atoms act as additional *surface ligands* to help REE coordination, as observed in surface organometallic chemistry.^65^ The functionalized sorbents show distinctive selectivity towards REE ions, depending on the positioning of the amide moieties (Figure 6). Specifically, the KIT‐6‐1,2‐PA sample exhibits the smallest *bite angle* and shows an impressive affinity toward late lanthanides, with a K_d_ value for Lu^3+^ as high as 54 000 mL g^−1^. In the case of the KIT‐6‐1,3‐PA material, with a larger *bite angle*, a well‐defined selectivity toward large‐/middle‐size lanthanides (i. e., Ce^3+^, Pr^3+^, Nd^3+^, and Sm^3+^) was observed, while no selectivity was observed for KIT‐6‐1,4‐PA due to the *para* positioning of the two carbonyl groups, preventing any synergistic action of the moieties that could have led to efficient sorption of lanthanide ions. This selectivity drastically changes from the homogeneous models that did not exhibit any selectivity, as expected. Furthermore, cycling tests of the sorbents performed in dynamic flow‐through systems showed reusability after 10 extraction‐stripping‐regeneration cycles without any significant loss in the extraction performance, which is of critical significance for the practical applications of such materials. In this case, compared to freshly prepared KIT‐6‐1,2‐PA, the recovered sorbent after the reusability test showed an increase in both surface area (from 726 to 768 m^2^ g^−1^) and pore size (from 7.0 to 7.3 nm), which may be explained by the leaching of some organic moieties attached to the surface of silica. Nevertheless, solid‐state NMR spectroscopy and the small mass loss change observed by TGA suggest that this increase is probably caused mostly by hydrolysis of residual ethoxide groups left on the silicon atoms of the organic precursors after grafting. Furthermore, when exposed to leachates of silicate and niobium mining deposits containing complex ionic matrixes, these functional sorbents showed also quite good selectivity, suggesting possible industrial applications.

**Figure 7:**
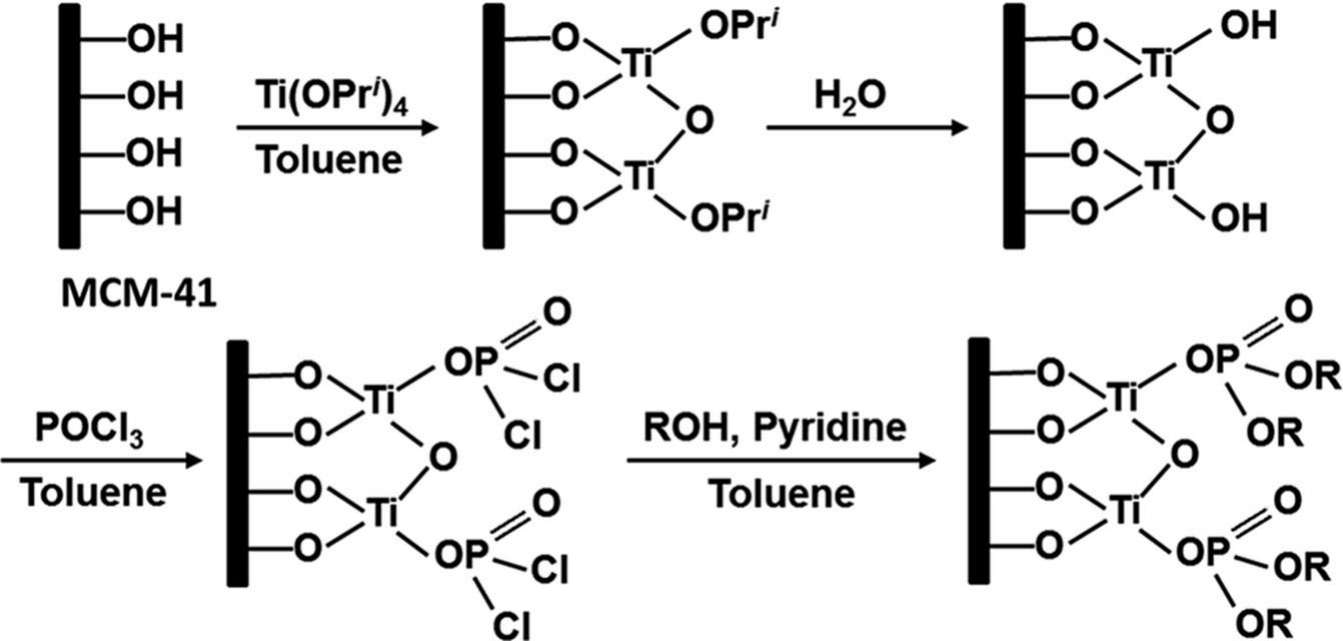
Layer‐by‐layer synthesis route of MCM‐41 silica functionalized with titanium(IV) *n*‐alkylphosphates (R=H, Et, *n*‐Pr and *n*‐Bu). Reproduced with permission from ref [66]. Copyright 2017, RSC.

Regarding the inclusion of selective ligand systems, Zhang *et al*. recently reported mesoporous MCM‐41 silica materials functionalized by titanium alkylphosphate using a solution‐based layer‐by‐layer deposition route for enhanced uptake of rare earth ions.^66^ The overall grafted structure was inspired by the LLE extractant tri‐*n*‐butyl phosphate (TBP) (Figure 2). The MCM‐41 silica was first grafted with a titanium(IV) phosphate generated *via* ‘acid‐base pair’ precursors Ti(O*i*Pr)_4_ and POCl_3_, followed by the grafting of alkoxide groups (ethyl, *n*‐propyl and *n*‐butyl) through a condensation reaction with the corresponding alcohols (Figure 7). Under optimized conditions, the hybrid materials showed a high uptake for Sc^3+^ from nitrate feed solutions, with a Sc^3+^‐ La^3+^ separation factor (SF) higher than 100 000 at pH 2.1. The SFs calculated for Dy^3+^‐ Nd^3+^ are approximately 3, comparable to that of TBP in the LLE system. Compared to the typical functionalization with organosilanes, the titanium(IV)‐phosphate acts as an acid‐resistant platform for the grafting of organic chains *via* a Ti(IV)−O−P bond, thus enhancing the tolerance of the sorbents towards acidic conditions.

Besides the above‐mentioned materials, several other SPE systems based on mesoporous materials using grafting procedures were recently reported for REE adsorption. Ligands such as diethylenetriaminepentaacetic acid (DTPA), diethylenetriaminepentaacetic dianhydride (DTPADA), *N,N*‐bisphosphono(methyl)glycine (BPG), ethylenediaminetetraacetic acid (EDTA), triethylenetetraminehexaacetic acid (TTHA), and phosphonoacetic acid (PAA) have been grafted on different nanoporous silica supports (e. g., silica gel^67^ and SiO_2_‐based nanoparticles^68^,^69^). These studies show that the selectivity of the resulting hybrid sorbents can easily be tuned by changing the ligand chemistry and geometry.

### 4.2 Ordered Mesoporous Carbons (OMC)

**Figure 8:**
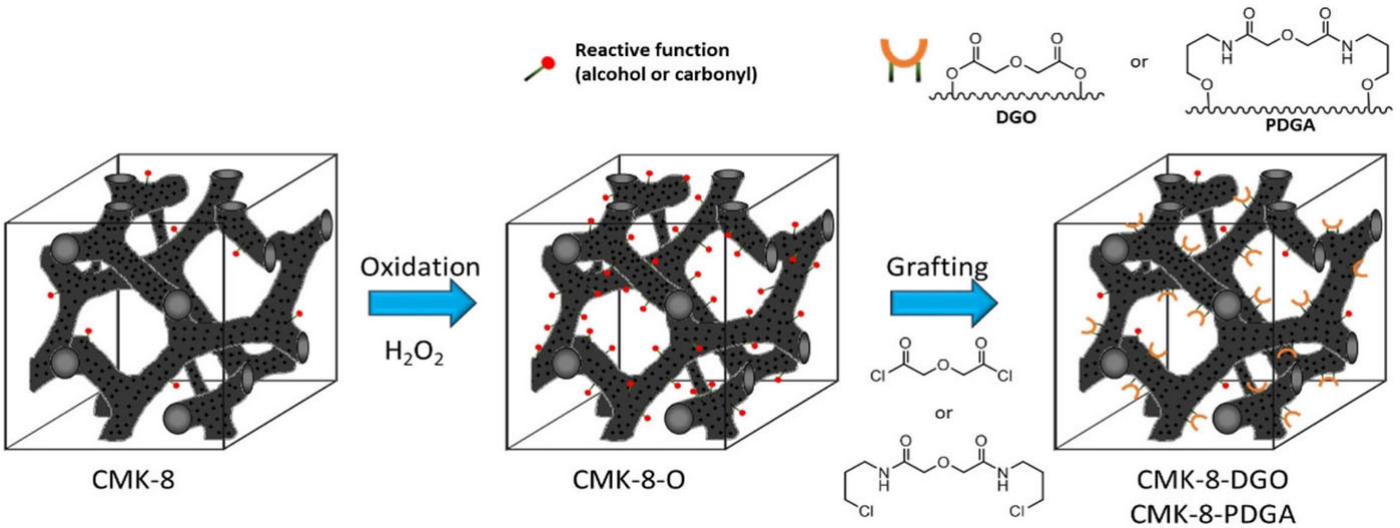
Schematic representation for the synthesis of the ligand‐functionalized mesoporous carbons. Reproduced with permission from ref [41]. Copyright 2017, ACS.

To overcome the relative low stability which can be associated with silica‐based functionalized sorbents in acidic conditions, ordered mesoporous carbons (OMC) could be interesting and viable alternatives since they present suitable porosity features and higher chemical resistance at lower pH. In 2014, Parson‐Moss *et al*. reported the potential of oxidized ordered mesoporous carbons as novel and effective scavenger for actinide and lanthanide cations.^70^ Nevertheless, effective surface functionalization of nanoporous carbons to anchor REE‐specific ligands has been a difficult task. To this aim, Lefrançois Perreault *et al*. described selectively functionalized mesoporous carbon materials with unprecedented affinity and adsorption capacity towards REEs under practical extraction conditions.^41^ The grafting procedure developed for the synthesis of new nanoporous sorbents leads to robust materials that can be reused, especially under the required acid conditions, thereby increasing their marketable value. In that study, CMK‐8‐type carbon exhibiting a 3‐D cubic mesopore network structure was chosen as a model of possible nanoporous carbon material. First, a wet‐oxidation technique was performed to increase the surface reactivity of the pristine CMK‐8 carbon, and in a second step, a surface modification using a DGA‐based ligand precursor was performed (Figure 8). Two types of ligands were tested: the first contained a short spacer (i. e., the grafting of diglycolylester to form CMK‐8‐DGO), and the second had a longer one, based on the *N,N’*‐bis‐chloropropyl diglycolamide structure (CMK‐8‐PDGA). This study showed first that the presence of oxidized moieties on the carbon surface (CMK‐8‐O, *via* post‐oxidation) results in a non‐specific adsorbent for lanthanides. Then, the two ligands with different anchoring moieties were both successfully grafted on the CMK‐8 surface; however, it demonstrated the necessity of highly reactive anchoring functionalities to do so, and a rigid ligand structure to achieve selective lanthanide extraction. At pH 2.6, a selectivity and high extraction capacity toward light lanthanides were observed with CMK‐8‐DGO. This material was also recycled up to 10 times. On the other hand, the overall extraction capacity and the selectivity were reduced significantly when the shorter spacer was replaced by the longer one (CMK‐8‐PDGA). It is worth noticing that although the K_d_ values for CMK‐8‐DGO are higher than that for the DGA‐modified mesoporous silica counterparts (e. g., KIT‐6‐DGA),^56^,^60^ which could be explained by the higher surface area of the mesoporous carbon, the selectivity is less pronounced in the case of CMK‐8‐DGO. For instance, the Gd^3+^‐ La^3+^separation factor is close to 4.5 for KIT‐6‐DGA, and only approximately 1.5 for CMK‐8‐DGO. This difference in selectivity could be due to the abundant amount of oxidized moieties on the carbon surface (CMK‐8‐O sample), and lower amount of functional groups grafted on the surface. In the case of the KIT‐6‐DGA hybrid material, a total mass loss of 24 % was observed by TGA,^60^ whereas less than 7 % of the mass loss could be attributed to the tethered DGO groups for CMK‐8‐DGO, due to the difficulty of surface functionalization *via* grafting. Nonetheless, this study suggests that the proposed surface modification of the CMK‐8 carbon could as well be adapted in the future to other (meso)porous carbons (e. g., biomass‐derived carbons),^71^ and ultimately lead to new types of sorbent materials for various analytical and industrial applications.

### 4.3 Ion‐Imprinted Mesoporous Materials (IMM) and Other Nanopores‐Related Strategies with Great Potential

**Figure 9:**
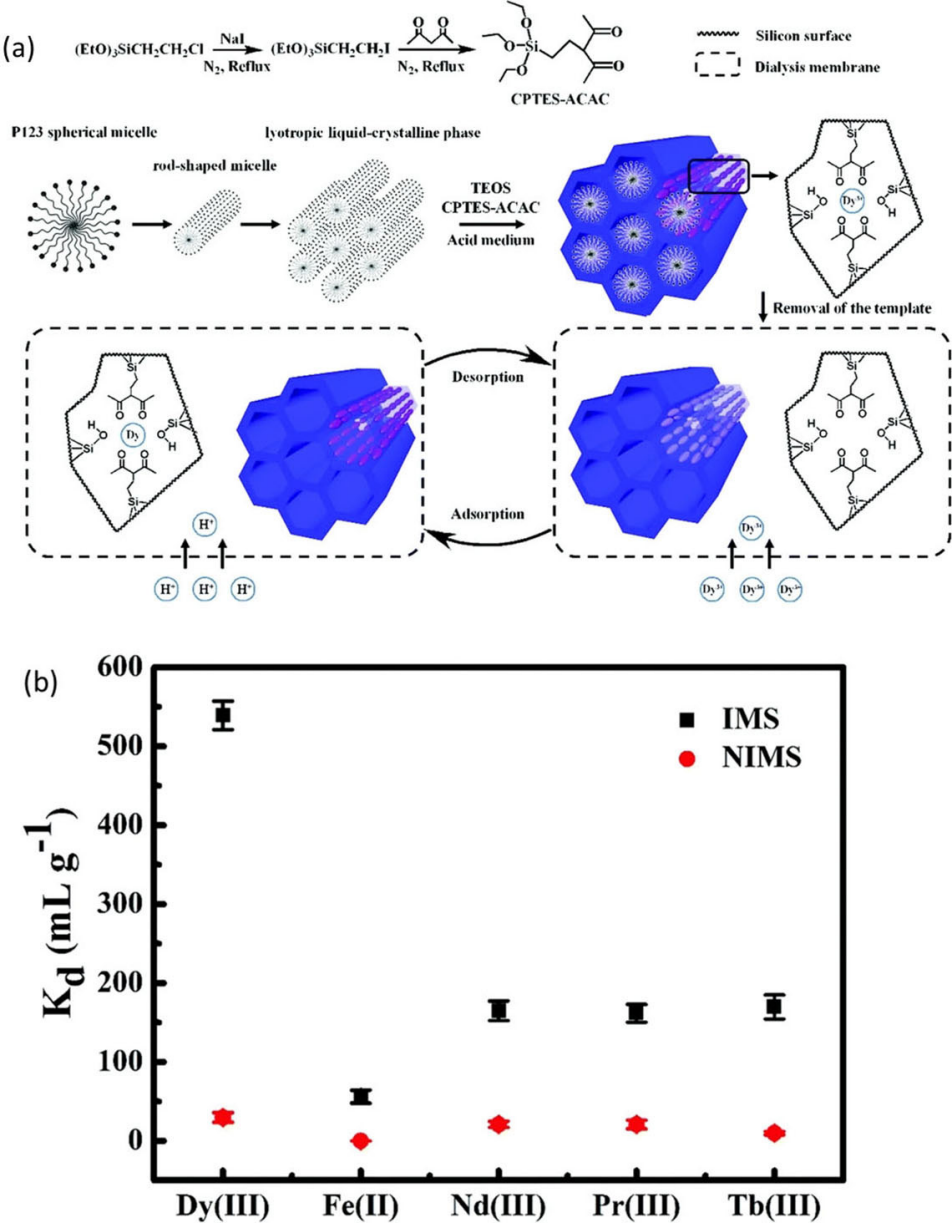
(a) Schematic of the imprinted mesoporous silica (IMS) and adsorption mechanism of Dy^3+^. (b) K_d_ values of the IMS and NIMS for a mixture of Dy^3+^, Fe^2+^, Nd^3+^, Pr^3+^, and Tb^3+^ (for acronyms, see text). Reproduced with permission from ref [76]. Copyright 2016, RSC.

In order to further improve the selectivity of sorbents, the ion‐imprinting technique (IIT) was introduced into the metal extraction/separation field. Its high selectivity stems from the affinity between organic ligands and templated ions, as well as the specific size of the templated‐shaped cavities. The IIT has been widely used for the synthesis of ion‐imprinted porous materials and polymers for the recognition of heavy metals and actinides;^72^, ^73^, ^74^, ^75^ therefore, its potential for REE adsorption has become obvious. In the work of Zheng *et al*., for example,^76^ highly ordered Dy^3+^‐imprinted SBA‐15‐type mesoporous silica modified with acetyl acetone (ACAC) was prepared for the recovery of Dy^3+^ in acid media. In this study, the ACAC ligand was first converted into an organosilane precursor, which was then complexed with Dy^3+^ and used in a one‐pot co‐condensation synthesis of the mesoporous hybrid material (Figure 9a). After removal of the template Dy^3+^ ions, the ion‐imprinted mesoporous silica (IMS) was sealed in a dialysis bag. The system shows high selectivity toward Dy^3+^ against competitive ions (Fe^2+^, Pr^3+^, Tb^3+^, and Nd^3+^) at pH 2 (Figure 9b), whereas the non‐imprinted equivalent material (NIMS) did not demonstrate such a selectivity. Along the same line, the same group also reported a system for the recovery of Nd^3+^ on the basis of biomass‐derived (cotton) mesoporous carbon films following a so‐called *dual‐template docking oriented* ionic imprinting procedure.^77^ There, cellulose nanocrystals were used as structure‐directing imprinting templates that complex Nd^3+^. In another recent example, Patra *et al*. investigated ion‐imprinted mesoporous carbon materials for the preconcentration and trace level detection of Gd^3+^ from complex matrices, in which soft‐templating (using Pluronic F127) was used to synthesize the carbon, with the addition of vinylsilane for the templated complexation with Gd^3+^.^78^ The materials thus‐obtained were then assembled in devices (fibers or cartridge) for solid phase microextraction (SPME) and micro solid‐phase extraction (μ‐SPE) studies. The SPME fibers showed a higher preconcentration factor (>1400 for Gd^3+^) with a detection limit down to 2.3 ng L^−1^, whereas the μ‐SPE cartridge showed a higher adsorption capacity (30 μg g^−1^) and removal efficiency (90 %) toward Gd^3+^. Both devices were also applied to the preconcentration, detection and removal of Gd^3+^ from real‐world samples, such as pathological laboratory wastewater, drinking water, and sewage sludge.

Besides the methodologies mentioned above, several emerging techniques connected to the presence of nanopores have demonstrated their potential for efficient and selective separation of REEs. For instance, functionalized core‐shell magnetic nanoparticles could be interesting candidates for the selective extraction of metal traces from mining wastes or industrial effluents. The magnetic (Fe_3_O_4_) cores provide the possibility to magnetically recover them from the aqueous phase, and the presence of nanoporous SiO_2_ or TiO_2_ shells facilitates the functionalization of the particles’ surface with organic ligands.^79^,^80^ Porous polymers have also been drawing attention as a new class of sorbents, owing to their strong and chemically resistant polymer network. Furthermore, the polymerization process allows the versatile functionalization of the material, depending on the monomer or cross‐linker utilized during the synthesis.^81^, ^82^, ^83^ Therefore, it is expected that such strategies could also be coupled and combined with some mesoporous components to build composites and/or hierarchical structures, which will eventually lead to highly efficient practical sorbents.

## 5 Summary and Outlook

Recently, significant advances have been made on the development of modern hydrometallurgical extractions procedures. Nanoporous materials, especially functionalized ordered mesoporous silica and carbon, have demonstrated their capacity as efficient and robust sorbents in SPE systems. Although LLE systems have inspired the choice of organic ligands used to functionalize nanoporous solid supports, several of our studies have demonstrated a significant difference in sorption/extraction behavior between LLE and SPE systems. Therefore, it is difficult to predict the extraction performance of the novel sorbents merely based on the coordination chemistry of the ligands, since the support surface and rigidity of the coordination environment creates unique cavities, similarly to host‐guest chemistry. In addition, the functionalization method used to synthesize hybrid materials also plays a crucial role in determining the overall performance of the sorbents. Different pathways or procedures (grafting in one‐step or two‐step, co‐condensation, or formation of PMOs) can be used to introduce organic moieties, depending the nature of functional groups or the extraction conditions envisioned. Then, to investigate the extraction performances and sorption mechanism, experimental batch techniques are widely used as an extremely convenient and efficient method. This approach can provide valuable information about the adsorption equilibrium time and kinetics, the maximum adsorption capacity (including isotherm studies), and selectivity.

**Figure 10:**
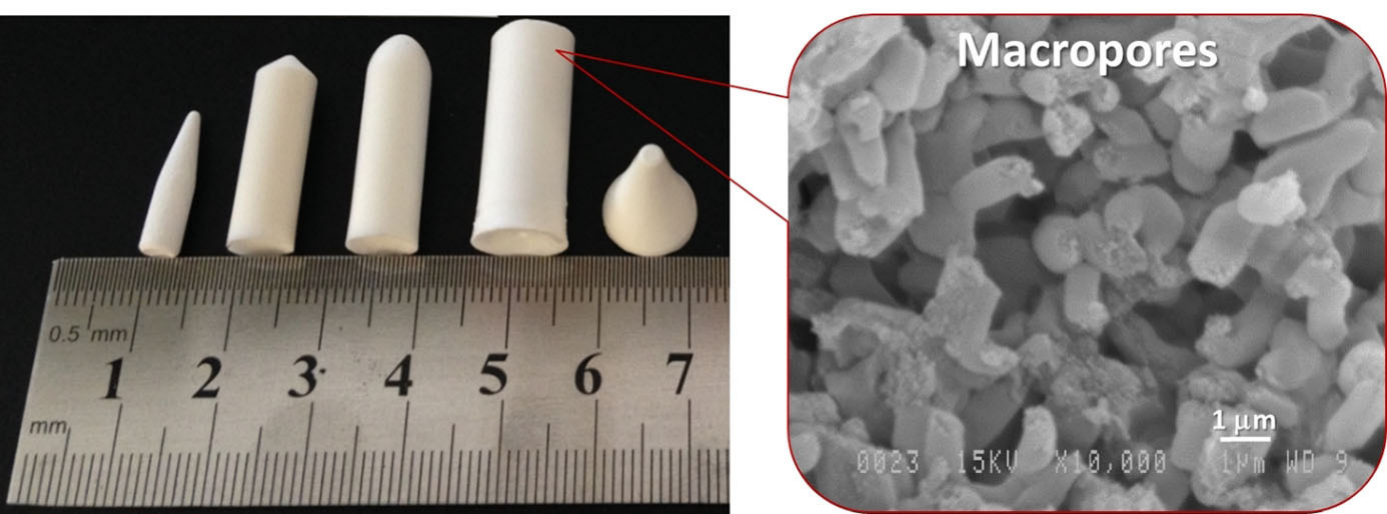
Photograph (left) of bimodal, mesoporous‐macroporous monoliths of different shapes, and a corresponding SEM image (right) showing the macroporous structure.

The selective separation and purification of REEs in an environmentally benign and cost‐effective way remain a challenging task, especially when it comes to the application of SPE systems under a real‐world scenario, such as extracting under dynamic conditions REEs from mining residues or end‐of‐life electronics. For instance, the permeability issue associated with column or cartridge systems is one of the biggest challenges to tackle before the OMMs can be applied into industrial applications, as the packing of mesoporous materials (in most cases as powders) into columns often results in a high back‐pressure. Therefore, our group is currently working on the development of functionalized hierarchically organized mesoporous‐macroporous (silica) monoliths, in which the mesopores provide high functionality while macropores facilitate mass transport. Processability of the materials to obtain specific shapes is achievable using sol‐gel synthetic methods. Such silica monoliths have been successfully synthesized with various shapes (e. g., columns, powder, fibers, disks, or capillaries), depending on the molds used (Figure 10). In principle, the surface of the monoliths can easily be functionalized with various selective ligands by simple post‐synthesis modification (grafting) or wet impregnation techniques. We expect that these new systems will be used as SPE materials and eventually implemented by the industry.

Last but not least, the potential of recycling of REEs from electronic waste (*e*‐waste) in order to ensure the supply as well as the efficient usage of REE resources is extremely attractive from an economical and environmental standpoint; however, only a few successful examples have been reported thus far. Therefore, further research should be devoted to this particular topic. In that perspective, ion‐imprinted mesoporous materials have shown to be quite specific/selective systems for individual REE sorption, and thus could be an effective approach for the separation and purification of a few targeted elements.
